# Modern Approaches for the Development of New Herbicides Based on Natural Compounds

**DOI:** 10.3390/plants12020234

**Published:** 2023-01-04

**Authors:** Alexander Berestetskiy

**Affiliations:** Laboratory of Phytotoxicology and Biotechnology, All-Russian Institute of Plant Protection, Pushkin, 196608 Saint-Petersburg, Russia; aberestetskiy@vizr.spb.ru

**Keywords:** chemical herbicides, phytotoxin, natural compounds, biorational herbicides, screening, formulations, synthesis, mechanisms of action

## Abstract

Weeds are a permanent component of anthropogenic ecosystems. They require strict control to avoid the accumulation of their long-lasting seeds in the soil. With high crop infestation, many elements of crop production technologies (fertilization, productive varieties, growth stimulators, etc.) turn out to be practically meaningless due to high yield losses. Intensive use of chemical herbicides (CHs) has led to undesirable consequences: contamination of soil and wastewater, accumulation of their residues in the crop, and the emergence of CH-resistant populations of weeds. In this regard, the development of environmentally friendly CHs with new mechanisms of action is relevant. The natural phytotoxins of plant or microbial origin may be explored directly in herbicidal formulations (biorational CHs) or indirectly as scaffolds for nature-derived CHs. This review considers (1) the main current trends in the development of CHs that may be important for the enhancement of biorational herbicides; (2) the advances in the development and practical application of natural compounds for weed control; (3) the use of phytotoxins as prototypes of synthetic herbicides. Some modern approaches, such as computational methods of virtual screening and design of herbicidal molecules, development of modern formulations, and determination of molecular targets, are stressed as crucial to make the exploration of natural compounds more effective.

## 1. Introduction

Modern agriculture faces a difficult task: to provide food to the world’s population with a minimal negative impact on the environment. The fight against weeds as a permanent component of anthropogenic ecosystems is essential for realizing the potential of agricultural crops. At high crop infestation, many technological elements (fertilization, use of highly productive varieties, plant stimulators, etc.) turn out to be practically meaningless due to enormous yield losses. According to the data of 2013–2014, yield losses of wheat caused by weeds in North America reached 22%, while in the absence of chemical control measures, the losses exceeded 35% [1]. In India, the weed infestation of peanuts led to a loss of 36%, soybeans—31%, corn—25%, and wheat—19% of the potential yield [2]. In this regard, the volume of use of chemical herbicides (CHs) worldwide significantly exceeds the volumes of other plant protection means [3]. However, the proportion of CHs used and their importance may vary depending on the protected crop [4].

In modern crop production systems, especially in developed countries, the paradigm of large-scale, intensive, mechanized agriculture with the use of a limited number of crops, as well as the intensive application of mineral fertilizers and chemical pesticides, primarily CHs, dominates. This has led to the contamination of groundwater with xenobiotics, inappropriate crop suppression, loss of natural vegetation, decrease in soil biodiversity, and negative impact on the health of farmers and consumers [5].

The use of active ingredients of pesticides, which are dangerous to human and animal health, has been prohibited. This stimulates the search for more active but less toxic CHs. Some highly toxic herbicides (for example, atrazine and paraquat (**7**)) are already prohibited, or a ban on their use (for example, glyphosate and glufosinate) is expected. At the same time, glyphosate-based herbicides occupy more than a third of the herbicide market in many countries [4], which creates a potential shortage of non-selective CHs.

Another serious problem of agriculture is the emergence of CH–resistant populations of weeds. Without adequate measures to bring to market new active ingredients with novel mechanisms of action and combined efforts to regulate the proper use of CHs, weeds can reduce global food production by 20–40% in the coming years, leading to a possible food deficiency with significant economic and social consequences. Unfortunately, the overuse of transgenic crops resistant to a number of herbicides and the consolidation of chemical companies has led to the fact that over the past 25 years, no herbicide with a fundamentally new mechanism of action has been introduced. The problem of the emergence of herbicide-resistant weeds is recognized as urgent as the issue of multiple antibiotic resistance in human pathogens [4].

Lately, the scientific problem of weed control has been getting into focus: in 2018–2022, an explosive growth of theoretical and experimental publications on the development of new CHs was noted. It is predicted that no more than 3–4 new molecular targets for CHs and the corresponding molecules acting on them are most likely to be identified until 2050, while at the same time, more biological and natural herbicides will be developed and widely used [6]. Taking this into account, the presented literature review addresses the problems of the development of natural or biorational herbicides (BCHs) based on unmodified natural products (NPs) as well as NP-derived (semi-synthetic NP derivatives) and NP mimic (synthetic NP analogs) herbicides and the approaches of their solution. It considers (1) the main current trends in the development and application of CHs; (2) the enhancement and practical application of NPs for weed control; (3) natural phytotoxins as prototypes of semisynthetic and synthetic CHs.

**Figure 1:**
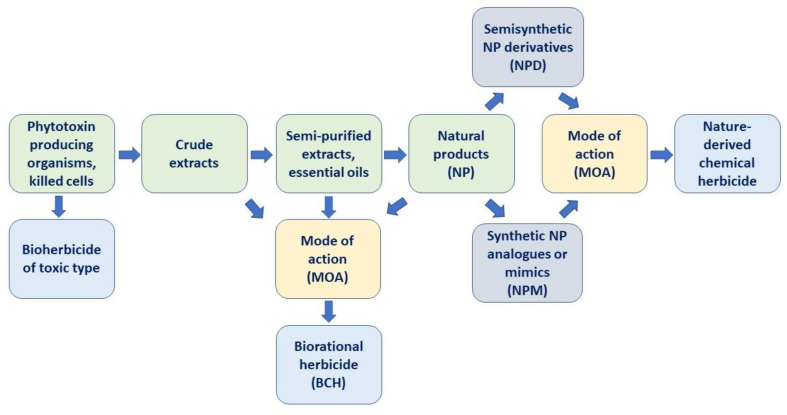
A scheme for the development of herbicides based on natural compounds.

The brief scheme of the development of chemical weed control means based on natural products is given in Figure 1.

## 2. Current Trends in Herbicide Development

Chemical herbicides are active ingredients (AIs) formulated for weed control, which should be characterized by (1) fast and high efficiency at low application rates over a wide temperature range; (2) a unique mechanism of action (to combat resistant species); (3) selectivity; (4) safety for non-target organisms; (5) low cost; (6) stability in the formulation; (7) rapid decomposition to non-toxic metabolites in nature [7]. The composition of a liquid or solid herbicidal formulations includes numerous auxiliary components and depends on the properties of AI, the method of application of the herbicide, target weeds, and protected crops [8].

Recently, there has been great progress in the screening techniques of herbicidal molecules, studying the mechanisms of their action, as well as in the methods of CH formulation.

**Figure 2:**
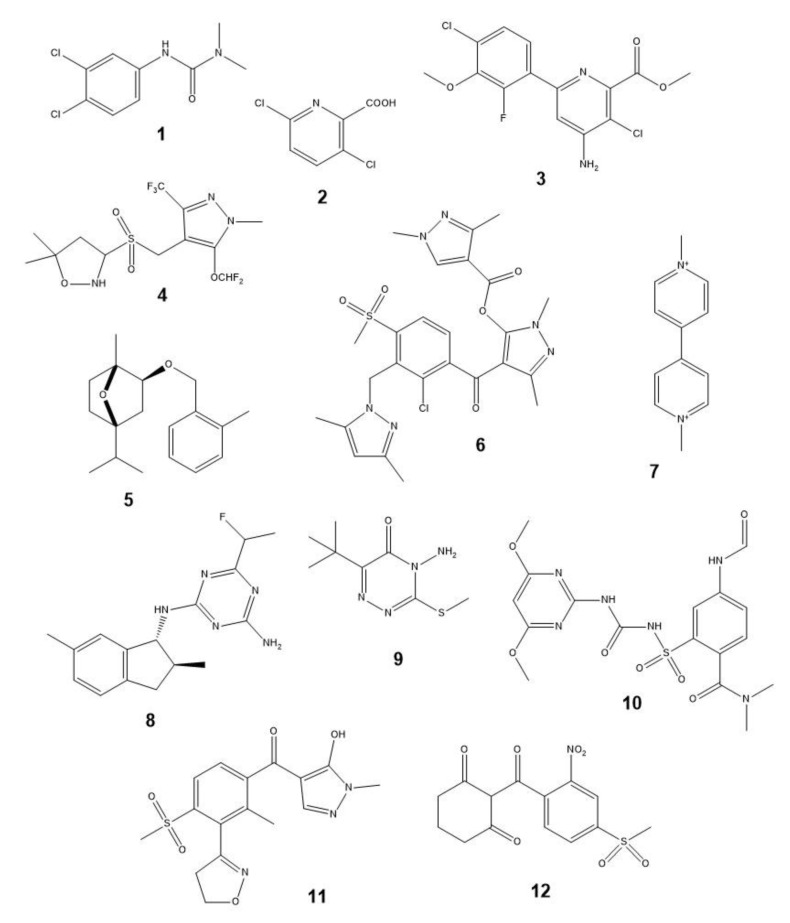
Examples of some common chemical herbicides: **1**—diuron, **2**—clopyralid, **3**—halauxifen-methyl, **4**—pyroxasulfone, **5**—cinmethylin, **6**—tripyrasulfone, **7**—paraquat, **8**—indaziflam, **9**—metribuzin, **10**—foramsulfuron, **11**—topramezone, and **12**—mesotrione.

Some AI structures of CHs discussed in the text are presented in Figure 2.

### 2.1. Screening

Two main algorithms are used to search for the herbicidal molecules: the classical “phenotypic” approach, based on determining the symptoms and the degree of phytotoxic activity caused by the tested substances, and the modern “directed” approach, based on the screening of a specific molecular target (MT) inhibitors in vitro. The algorithm of the phenotypic approach research is as follows: (a) detect a chemical substance with phytotoxic activity in vivo; (b) recognize the mechanism of action (MOA)/MT as a known or unknown class; (c) set MT if the substance belongs to a previously unknown class; (d) optimize the chemical structure to produce a commercial product in accordance with the above requirements for CH. The algorithm of the directed approach includes the following steps: (a) select the target protein (MT); (b) reveal substances with inhibitory activity against MT in vitro; (c) improve their chemical structure to achieve an activity in vivo; (d) optimize the structure of CH to obtain a commercial product [9].

According to the general estimates, it is necessary to analyze at least 150,000 substances to identify the lead compound [10]. In this regard, labor-intensive in vivo phytotoxicity bioassays in greenhouse or controlled conditions are usually conducted at the final stages of the screening research. To accelerate and optimize the selection of substances from various libraries for the bioassays, the computational methods based on the analysis of physico-chemical properties and molecular docking (if a known MT is assumed) of substances are used; after that, the high-throughput screening in vitro could be used to validate the selection.

The source of information for virtual screening commonly includes chemical structures from published materials or available databases and any commercial or original libraries of synthetic and/or natural compounds. As a rule, specialized sets of drug-like substances are used [11] since some of them possess phytotoxic properties, for example, sulfonamide antibiotics [12], salicylic acid [13], hypotensive [14], and antimalarial substances [15], as well as statins [16]. There are also specialized commercial libraries of herbicide-like compounds [17,18].

The following approaches are common to establishing original libraries of synthetic compounds: (1) the use of products of combinatorial chemistry; (2) the “me too” approach—the synthesis of new molecules based on the structures of known herbicides, (3) “typical toxophores” approach—the use of a “standard” set of structural fragments that increase the effectiveness of herbicidal molecules [19], usually halogen-containing ones [20,21]. The extracts from natural sources (bacteria, fungi, and plants) are also a kind of library of bioactive substances from which new phytotoxic compounds can be isolated.

#### 2.1.1. Virtual Screening

The computational methods of chemo- and bioinformatics, approved for the development of new drugs, are employed to search for herbicidal molecules. The analysis of physico-chemical properties of existing CHs and their mobility in plants allowed to propose a number of predictors for the selection of promising molecules: molecular weight (200–450 Da), lipophilicity (log *P* 1–5), number of acceptor groups (1–9) and hydrogen bond donors (0–2), number of rotating bonds (1–8), and some other parameters [22,23,24]. In general, the similarity of the physico-chemical properties of herbicides and antimalarial drugs has been shown [15]. Databases and machine learning methods are being developed for the virtual evaluation of any molecules as potential CHs [25,26]. Computational methods able to predict the overall toxicity of certain molecules, such as PASS software [27], including herbicidal compounds [28,29], have been developed. Software for the virtual MOA identification of novel phytotoxic molecules has been created [30]. If the crystal structure of the target protein was established, the selection of optimized herbicidal molecules with maximum affinity to MT can be performed on the basis of molecular docking. After the virtual selection of promising molecules, the validation of their phytotoxic properties in vitro and/or in vivo bioassays is necessary.

#### 2.1.2. Bioassay Techniques

For the rapid evaluation of a large number of substances (taking into account their relatively high cost), high-throughput screening techniques are widely used, which are commonly carried out in 24- or 96-well plates. Typically, the specified samples of compounds dissolved in water, acetone, or dimethyl sulfoxide (or another solvent) are added to the nutrient substrate for algae cultivation or for the germination of plant seeds. After 1–7 days of incubation, the optical density of cell cultures is determined, or seed germination level is observed [15,31].

For screening phytotoxic substances by measuring the length of seedling roots and shoots, a bioassay using lettuce (*Lactuca sativa* L.) seeds seems to be most reproducible [32]; however, a strict protocol should be followed [33]. A highly sensitive bioassay with mustard (*Sinapsis alba* L.) seeds was established for the evaluation of phytotoxic allelochemicals [34]. Among the monocot plants, the use of *Agrostis stolonifera* L. and *Alopecurus myosuroides* Huds. seeds [35,36], as well as several species of aquatic macrophytes of the genus *Lemna* (e.g., *L. paucicostata* Hegelm., *L. minor* L., *L. gibba* L.) [37,38], was adapted for bioassays. In the screening programs conducted by Chinese researchers, *Brassica campestris* L., *Cucumis sativus* L., *Amaranthus retroflexus* L., *Digitaria sanguinalis* (L.) Scop., *Echinochloa crus-galli* (L.) P. Beauv. are preferred for seed germination tests performed in Petri dishes [39]. *Arabidopsis thaliana* (L.) Heynh, which can be germinated and cultivated in 96-well plates on Murasige Skuga medium with 0.1% agarose [15,40], is also useful for high-throughput screening. The growth inhibition bioassays with algae *Chlorella pyrenoidosa* H. Chick. [41] and *Chlamydomonas reinhardtii* R.W. Howshaw and H. Ettl [42] allow to evaluate a number of additional physiological parameters, such as photosynthesis inhibition [43,44].

Numerous techniques have been developed for the targeted screening of herbicidal molecules. For example, a technique for assessing the “acute” phytotoxicity of the herbicide diuron (**1**) was established with detached *Halophila ovalis* (R. Brown) Hooke leaves in 12-well plates using chlorophyll fluorescence analysis [45]. A technique of the fluorescence analysis for high-throughput screening of CH that cause rapid peroxidation of plant membranes is proposed based on the fact that peroxide in the cell fluid can be determined with a fluorescent compound, which is formed as the result of the reaction of homovanillic acid and peroxidase [46]. For the identification and characterization of C4 photosynthesis inhibitors in vivo, a technique based on the measurement of oxygen release into the medium by a suspension culture of chlorenchyma cells obtained from the C4 plant, *Bienertia sinuspersici* Akhani, was developed [47]. A fluorescent platform for the targeted in vivo screening of 4-hydroxyphenylpyruvate dioxygenase (HPPD) inhibitors was proposed. The improved fluorescent label visualizes HPPD in living cells and in Danio fish for real-time direct monitoring of this enzyme inhibition [48].

### 2.2. Mechanisms of Action and Molecular Targets of Herbicides

The separation of the active substances of CHs by their MOA or MT makes it possible to plan their rotation and reduce the risk of resistance in weeds. At present, the knowledge of MOA of herbicides is a fundamental aspect of their development [49]. So far, 26 herbicidal MTs are known to be toxic to plants due to the disorders of (1) photosynthetic processes, (2) critical metabolism stages, and (3) normal growth [3].

To combat the weed populations resistant to widely used CHs, new MTs are needed. It is interesting to note that for a number of well-known herbicidal molecules, MOAs are still being elucidated. In order to diagnose MOA of new herbicidal substances selected as a result of phenotypic screening, the symptoms of plant damage are evaluated, and a number of physiological and biochemical tests are conducted. The results of the tests may be characteristic of CHs with known MOA. The standard protocols were proposed for the validation of MT diagnosed by “physionomics” [50,51]. Further elucidation of novel MTs may be conducted with metabolomic [52,53], transcriptomic [54], and genomic methods [55]. The most promising way to search for new MTs and their inhibitors is to identify and evaluate the quantitative content of certain key proteins/enzymes in plant cells, as well as to study the mechanisms of resistance of organisms to CHs or natural phytotoxins [9,56,57].

The combination of physiological, biochemical, and omics approaches allowed to establish MT for pyroxasulfone (**4**) [58] and many other herbicides [53]. MOA and MT are being clarified for AIs of already introduced or even banned CHs, such as phenol derivatives [59] and glufosinate [60]. Fatty acid thioesterase, which participates in the pathway of plant lipid biosynthesis, releasing fatty acids from acyl-transferring proteins in plastids, which is necessary for their subsequent transfer to the cytoplasm and endoplasmic reticulum, was recently identified as a target of cinmethylin (**5**), commercialized back in the mid-1980s [61]. Homogentisate-solanesyltransferase, a recently discovered herbicidal MT, catalyzes the decarboxylation and prenylation of homogentisate to form 2-methyl-6-solanesyl-1,4-benzoquinol during the biosynthesis of plastoquinone, which in turn is involved in the biosynthesis of carotenoids as a cofactor of phytoene desaturase. Mitsui Chemicals Agro, Inc. (Tokyo, Japan) recently released a selective herbicide cyclopyrimorate (**45**) against rice crop weeds acting on this MT [62].

The CHs with new MOAs, as recently considered, will not become a panacea against multiple CH-resistant and highly evolutionary weeds. However, substances affecting two or more MTs may retain their high herbicidal properties for a relatively longer period [63,64,65]. A rational design of the structure of such dual inhibitors is possible. For instance, (Z)-2-(5-(4- methoxybenzylidene)-2,4-dioxothiazolidine-3-yl)acetic acid was found to inhibit two different enzymes of lysine synthesis in *A. thaliana*: dihydrodipicolinate synthase and dihydrodipicolinate reductase [66].

A search for more effective inhibitors of already known MTs by means of structural modification of widely used herbicidal molecules or screening of substances with fundamentally new chemical skeletons is underway. Two new auxin-like herbicides, Rinskor (florpyrauxifen-benzyl) (**44**) and Arylex (halauxifen-methyl) (**3**) were recently introduced [67,68]. The MT of the latter was suggested to be the auxin receptor that disrupts the homeostasis of indolyl-acetic acid and stimulates the over-synthesis of ethylene and abscisic acid, inducing overexpression of 1-aminocyclopropane-1-carboxylate synthase and 9-*cis*-epoxycarotinoid dioxygenase [69]. Indaziflam (**8**), the active ingredient of two new alkylazine herbicides, Specticle^TM^ and Alion^TM^, inhibits cellulose biosynthesis in plants [70]. Tripyrasulfone (**6**), a new CH belonging to the pyrazolones, was registered in 2020 for the control of annual weeds in rice fields; its MT being HPPD, as in the case of triketone CHs [71].

Some MTs look promising for the targeted development of novel CHs. For instance, a transketolase involved in the biosynthesis of lipids, amino acids, and nucleotides in plant cells has recently been used to model potential herbicidal molecules in silico: about 40 lead compounds have been synthesized, and more than half of them have shown promising herbicidal activity against rape (*B. campestris*) and barnyard grass (*E. crus-galli*) [72]. Inositol phosphoryl ceramide synthase, which is involved in the biosynthesis of plant sphingolipids, has been identified as a promising MT for the search for herbicidal molecules. A strain of yeast fungus, *Saccharomyces cerevisiae,* expressing an AtIPCS2 isoform of this enzyme from *A. thaliana* and showing sensitivity to its inhibitors, has been constructed. This strain was used to screen a library of more than 11,000 compounds provided by Bayer AG. The screening results were then validated in an in vitro enzyme inhibition test. This two-stage screening revealed a strong enzyme inhibitor that demonstrated selectivity against AtIPCS2 compared to the orthologous yeast enzyme and phytotoxic activity against arabidopsis seedlings [73].

### 2.3. Formulation of Herbicides

The formulation types of CHs can be solid (powders, granules, or microcapsules) or liquid (true or colloidal solutions in water or in organic solvents, emulsions, and suspensions). Beyond the active ingredients, the formulations consist of auxiliary components, for example, wetting agents, penetrants, solvents, buffers, microbiocides, adhesives, UV protectors, defoamers, and others (for example, inert fillers, dyes, and odorants). These additional components may be important to increase the stability of herbicides during storage and their effectiveness in the field [8]. The types and compositions of the formulations are constantly being improved in order to make the use of pesticides more effective, convenient, and as safe as possible [74].

The main trends in the development of the formulations of pesticides are as follows: the combination of several active ingredients in a single formulation; the use of aqueous emulsions or microemulsions with minimal volumes of organic solvents; the replacement of dusting powders with suspension concentrates or water dispersible granules; the increase in the pesticides targeting using slow release formulations or by seed coating; the search for adjuvants to increase biological activity and to reduce the application rates [75]. Thus, (1) the development of synergistic compositions of various active ingredients, (2) the search for adjuvants that increase the effectiveness of post-emergent CHs, (3) the development of granular slow-release formulations of pre-emergent CHs, as well as (4) the use of nanotechnology for CH formulations will be briefly discussed below.

The combined effect of 24 herbicides with different MOAs was studied with high-throughput screening on arabidopsis seedlings to demonstrate several new synergistic mixtures along with the known combinations (mesotrione (**12**)—atrazine and atrazine–clomazone). One component of newly identified synergistic CH combinations necessarily belonged to leaf-bleaching herbicides: mesotrione–norflurazone, mesotrione–kletodim, and clomazone–paraquat (**7**) [76]. In South Africa, resistant populations of *Plantago lanceolata* L. that are 10 and 20 times less sensitive to glyphosate and paraquat, respectively, than the wild plants were found. To effectively control them, a combination of terbutylazine and S-metolachlor (497.2 g/ha + 102.8 g/ha) with different MOAs was selected [77]. An experimental study of herbicide resistance in various populations of *A. myosuroides* has shown that the use of CH mixtures can contribute to the development of a multiple resistance mechanism in weeds [78]. Indeed, the number of weed populations insensitive to CHs with various MOAs (from 2 to 8 and more) is steadily growing [79,80]; in the future, both more accurate selection of active ingredients and more active use of agrotechnical and biological methods of weed control will be required [81].

Adjuvants are any useful substance added either to the formulation or to the spray solution to increase the phytotoxic activity of CHs through improved tank mixing, increased drip coating of the leaf surface, stronger droplet retention, and slower evaporation, facilitated penetration through the leaf cuticle, etc. [82,83,84,85]. Notably, in some cases, adjuvants can significantly change the toxicological profile of CHs [86]. Instead of synthetic components, less toxic surfactants of natural origin, for example, various lipids, were proposed as adjuvants [87]. Depending on the anatomical features of target weed species and crops, as well as the physico-chemical properties of CH, optimal adjuvants and their concentrations can be selected from extensive catalogs [88]. There are several examples below.

The laboratory and greenhouse evaluation of diuron (0.075 kg/ha) combined with 12 adjuvants (0.1% by volume) for control of monocot weeds showed that organosilicon surfactants reduce the surface tension and contact angle of droplets to a greater extent than the non-silicone surfactants in various spray solutions. Three selected organosilicon adjuvants allowed the herbicide to significantly suppress the growth of *E. crus-galli* compared with diuron alone [89]. The efficacy of foramsulfuron (**10**) against a number of weeds (*E. crus-galli*, *Setaria faberi* Herrm., and *Abutilon theophrastii* Medik.) was significantly increased when it was applied together with methylated vegetable oil (MSO Concentrate, Loveland Products, Inc., Greenville, MS, USA) compared to a nonionic surfactant or vegetable oil. Moreover, in some treatments, the addition of nitrogen fertilizer (ammonium nitrate or ammonium sulfate) to the spray solution further increased the effectiveness of the herbicide in the field [90]. The methylated vegetable oil is known to reduce the surface tension and the contact angle of droplets but also to prevent their drying, which provided a higher level of penetration of CH into plant tissues, as was shown by the example of topramezone (**11**) and GY-HMax methylated soybean oil on several weed species [91]. Ammonium fertilizers, together with other adjuvants, also contribute to more efficient absorption of some herbicides [82,84].

After the right selection of adjuvant, the application rate of CH can be considerably reduced. For instance, the nonionic adjuvant INEX-A (Cosmocel, Monterrey, Mexico), which is a mixture of polyglycols of ethoxylated alcohols and aryl-polyethoxyethanol, showing water-retaining, penetrant, and dispersant properties, allowed to reduce the application rate of glyphosate below the conventional levels and to suppress the growth of biomass of two weeds, *Lolium rigidum* Gaudin, and *Conyza canadensis* (L.) Cronq. Moreover, the adjuvant promoted faster uptake of the CH and its translocation in the plants [92]. Taking into account the chemical diversity of adjuvants, the selection of their appropriate brands and concentrations is a time-consuming and challenging process. In order to accelerate adjuvant selection, an express technique based on the determination of chlorophyll fluorescence of treated leaves has been developed [93].

Controlled-release formulations (CRFs) of CHs are usually water-insoluble granules that are physically, chemically, and/or biologically decomposable. CRFs consist of a polymer carrier (for example, lignin, polysaccharides, polyesters) and various additives allowing them to gradually release CH into the environment. CRFs protect active ingredients from the harsh effects of external factors and are less dangerous for handling. At the same time, CRFs prevent CH from entering the groundwater [94]. Recently, the development of CRFs has become a trend in agrochemistry [95].

Depending on the materials and techniques used for CRF preparation, CHs are obtained in the form of particles and capsules, which differ both structurally and in composition. The capsules are systems consisting of a polymer shell and a core, in which AIs can be dissolved or adsorbed on the polymer wall [96]. The particles (granules, microspheres) consist of a matrix in which AIs are either adsorbed or physically retained [97].

Oxyfluorophene encapsulated in polyurea was stable and nontoxic for rice [98]. Pendimetalin was successfully encapsulated in polyurethane urea; the composition of the shell significantly influenced the shape of microcapsules and the release of the AI [99]. The granular CRFs of metribuzin (**9**), tribenuron-methyl, and phenoxaprop-p-ethyl based on a mixture of biodegradable bacterial poly-3-hydroxybutyrate and natural materials (clay, wood flour, and peat) have demonstrated high stability, biological efficacy, and low side toxicity. The soil application of the CRFs was more effective than spraying [100,101,102]. Similar results were obtained using microcapsules based on poly(3-hydroxybutyrate-co-4-hydroxybutyrate) for controlled release of trifluralin [103].

In the last decade, the achievements of nanotechnology in the development of pesticide formulations have been actively discussed. This mainly applies to nanoparticles, the nanoscale analogs of CRFs [104]. Nanoformulations may include the components that release AI under certain conditions, for example, at a given pH level, illumination, temperature, and humidity, as well as in the presence of certain plant enzymes [105]. Nanotechnology also makes it possible to create the liquid forms of CHs (for example, nanoemulsions) and nanoadjuvants, for example, based on biosurfactants [106]. It is assumed that the use of nanomaterials in CH formulations will increase their biological efficiency by improving the absorption and translocation of AIs in plants [107]. However, due to the increased penetrating power and higher biological activity, the question of the safety of nanopesticides for humans and the environment is open [108,109,110].

Chitosan-tripolyphosphate nanoparticles loaded with paraquat had a stronger inhibitory effect on spinach photosystem I than the herbicide in a commercial formulation [111]. To prevent rapid droplet evaporation and/or leakage of hydrophilic herbicides into wastewater, alginate nanoparticles were synthesized in sunflower oil to encapsulate dicamba using a reverse mini-emulsion matrix. The resulting nanohydrogel with a particle size of about 20 nm provided the steady and prolonged release of the AI for ten days [112]. Atrazin and metribuzin nanoencapsulated into polycaprolactone were stable and more phytotoxic than their conventional formulations [113,114]. A biodegradable and photosensitive amphiphilic polymer was synthesized by polyesterification and was used to manufacture nanoscale particles with CH-controlled release triggered by light. The polymer nanoparticles loaded with 2,4-D were stable without UV treatment, while the AI was released from nanoparticles under ultraviolet radiation. Laboratory experiments have shown that this nanoCRF of 2,4-D exhibit a good herbicidal effect and reduced toxicity against non-target organisms [115].

## 3. Perspectives of Natural Compounds as Biorational Herbicides

### 3.1. Promising and Commercialized Natural Compounds

In order to reduce the amounts of synthetic CHs used for weed control, biorational (biochemical) herbicides (BCHs) are being developed in various countries, especially for organic farming [116,117]. Microbial pesticides of toxic action, crude extracts of plant or microbial origin, and formulations of individual natural compounds (or their mixtures) may be considered as BCHs [118,119] (Figure 1). The search for potential AIs for BCHs is typically based on the chemical ecology approach: plants can produce phytotoxic metabolites to compete with other species, while microorganisms can employ them for plant colonization [120,121,122,123].

**Figure 3:**
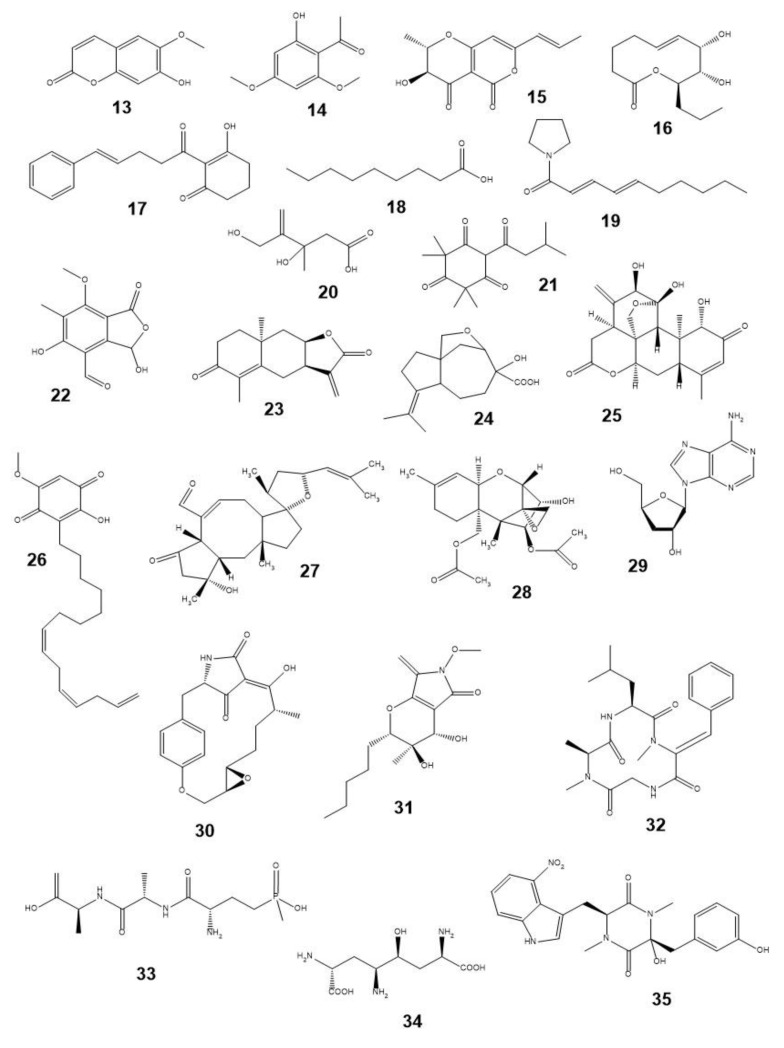
Structures of phytotoxins mentioned in the text: **13**—scopoletin, **14**—xanthoxyline, **15**—radicinin, **16**—herbarumin I, **17**—alatanone A, **18**—pelargonic acid, **19**—sarmentine, **20**—mevalocidin, **21**—leptospermone, **22**—cyclopaldic acid, **23**—inuloxin, **24**—aspterric acid, **25**—ailanthone, **26**—sorgoleone, **27**—ophiobolin A, **28**—diacetoxyscirpenol, **29**—cordycepin, **30**—macrocidin A, **31**—phaeosphaeride A, **32**—tentoxin, **33**—bialaphos, **34**—ascaulitoxin aglycone, and **35**—thaxtomin A.

Some examples of phytotoxins, which are discussed below, are presented in Figure 3. It also demonstrates that they belong to various classes of natural compounds [124,125].

Many natural phytotoxins display selectivity or high toxicity against problematic weeds such as parasitic species. The cinnamic acid methyl ester was found to suppress selectively annual ryegrass (*L. rigidum*) in wheat [126]. The fungal phytotoxin radicinin (**15**) demonstrated the selectivity against the buffel-grass (*Cenchrus ciliaris* L.), an invasive plant of the USA, and the absence of teratogenic, toxic, or lethal effects on *Brachydanio rerio* fish embryos [127]. An aqueous extract from mugwort (*Artemisia vulgaris* L.) suppressed the growth of the redroot pigweed (*A. retroflexus*) and stimulated the growth of maize [128]. A number of trichothecene toxins of *Fusarium* fungi at very low concentrations inhibit the seed germination of parasitic weeds. The most active toxin, diacetoxyscirpenol (**28**), completely suppressed the germination of *Striga hermontica* (Delile) Benth. and *Orobanche ramosa* L. seeds at concentrations <1 µM [129,130]. Some natural compounds (for example, fusicoccin, strigolactones, and their derivatives), on the contrary, stimulate their seed germination in the absence of a host plant, which leads to the death of the parasites [131,132].

The list of natural compounds exhibiting phytotoxicity that exceeds the activity of reference herbicides in laboratory experiments is constantly expanding. For example, cordycepin (**29**) (a well-known metabolite of the fungus *Cordyceps militaris*) at a concentration of 40 µg/mL suppressed the growth of radish (*Raphanus raphanistrum* L.) roots several times more effectively than glyphosate [133]. Several α-pyron derivatives from *Alternaria brassicicola* culture demonstrated herbicidal effects superior to glyphosate activity [134]. Fungal herbarumin I (**16**) inhibits the growth of *Amaranthus hypochondriacus* L. roots more strongly than 2,4-D [135], and it is also phytotoxic when assayed on punctured leaf discs of various plants [136]. Trichothecene toxins, harzianum A and harzianum B, isolated from the culture of the fungus *Trichoderma harzianum,* suppressed the germination of *E. crus-galli* seeds more effectively than the above-mentioned herbicide [137]. Some saponins from agave in concentrations <60 µg/L demonstrated noticeable allelopathic properties, and they were more active against *E. crus-galli* compared to the commercial post-emergence triasulfuron-based herbicide Logran [138]. Tanshinone I and tanshinone IIA from *Salvia* spp. biomass suppressed the growth of duckweed (*L. paucicostata*) by 50% at concentrations of 113 and 140 µM, respectively, at a level of phytotoxicity comparable to some commercial herbicides [139]. Sesquiterpenoids isolated from the leaves of perennial sowthistle (*Sonchus arvensis* L.) showed selective phytotoxic activity against redroot pigweed and fat hen (*Chenopodium album* L.) at the level of triasulfuron without affecting the wheat [140].

The MT of some natural phytotoxins was identified to show their unique MOA. Thus, their use could contribute to the control of CH-resistant weed populations [119,141]. Pyrenophorol was proposed as a selective herbicide for the control of winter wild oat (*Avena sterilis* L.) [142] with MOA differing from the action of the widely used CHs, such as glyphosate, mesotrione, norflurazone, paraquat, and diuron [143]. Thaxtomin A (**35**) produced by *Streptomyces scabies* was shown to interfere with the normal course of cytokinesis in onion root cells by inhibiting cellulose biosynthesis [144,145]. Ascaulitoxin aglycone (**34**), isolated from the culture of the fungus *Ascochyta caulina,* affects the metabolism of amino acids in sensitive plants by inhibiting aminotransferases [146]. Sarmentine (**19**), isolated from *Piper* spp., was proved to be promising as a BCH because it has several MOAs on plants (inhibits both photosynthesis and enoyl-acyl-transferring protein reductase) and also penetrates well through the leaf cuticle of a number of weeds [147]. Possible MTs of macrocidin A (**30**) of *Phoma macrostoma* are two enzymes, phytoene desaturase, and 1-deoxy-D-xylulose reductoisomerase, involved in the biosynthesis of carotenoids [148,149]. Aspterric acid (**24**), formed by the fungus *Aspergillus terreus*, inhibits the plant dehydratase of dihydroxy acids [56]. Tenuazonic acid blocks photosystem II by inhibition of D1 protein (*psbA*) with a binding site different from the one of diuron [150]. Recently, a number of mycotoxins, such as patulin, gliotoxin, and cytochalasin A, have also been found to bind to the D1 protein [44,151,152]. Cyclopaldic acid (**22**) from the fungus *Diplodia cupressii* was found to elicit immune response reactions in *A. thaliana* cells [153]. In the review [154], numerous plant disorders caused by plant essential oils and their components were listed.

Most commercial BCHs based on unmodified natural compounds mainly target organic agriculture and are costly products with a short shelf life, which require high application rates and repeated use [119,155,156]. A number of the strains of the bacteria, *Streptomyces hygroscopicus,* and *S. viridochromogenes*, synthesizes bialaphos (**33**) in culture. This BCH is produced in Japan, but it has a very limited market [157,158]. Several companies offer BCHs based on pelargonic acid (**18**) for use in organic farming, for example, Katoun Gold^®®^ produced by Belchim Crop Protection (Belgium) [159]. The company “Marrone BioInnovations” (USA) developed and registered the Opportune^TM^ herbicide. It is based on killed *Streptomyces acidiscabies* cells, and its active ingredient is phytotoxic thaxtomin A [119,160]. The reports on satisfactory Opportune efficacy seem to be limited. However, its synergistic mixtures with other NPs, such as manuka oil, could affect weeds more effectively [161]. Thaxtomin A formulations were shown to control some weed species, but several common weeds, such as perennial ryegrass or chickweed, were insensitive at their application rate of 380 g/ha [162,163].

Due to the high demand for BCHs for “inorganic” crop production, new, more effective herbicidal compounds of natural origin are being sought. In the former “Dow AgroSciences” company, a number of microbial phytotoxins promising for the development of new BCHs were identified: macrocidin A [164], cinnacidin [165], albucidin [166], mevalocidin (**20**) [167]. The “Marrone BioInnovations” company tested >12,000 microorganisms and prepared three BCHs for registration [168]. It has been reported that more than ten formulations based on plant extracts and essential oils were tested, but they have not been widely used [116,169]. The experimental BCH formulation containing tenuazonic acid as AI was successfully evaluated in the field against the main weeds of cotton and tobacco [170].

### 3.2. Enhancement of Biorational Herbicides

There are a number of problems in the commercialization of natural products (NPs) as BCHs. First, the results of in vitro and in vivo bioassays do not always correlate because of the difficult penetration of natural phytotoxins through plant cuticles. Moreover, different bioassays are often used in the evaluation of synthetic herbicidal molecules and natural compounds [171].

Second, techniques of NP mass production and BCH quality control have not been sufficiently developed, so they have rarely been evaluated in greenhouse and field conditions. Taking into account the generally low yields of NPs (1–100 ppm), scaling up the production of phytotoxins to the pilot or industrial level requires the selection of the best producer strain, optimization of nutrient medium and fermentation conditions, and development of a simple purification method with minimal use of organic solvents [172]. Working with plant metabolites, it is necessary to organize plantations of producing plants. Despite the above difficulties, recently, there have been examples of studies on optimizing the production of promising microbial phytotoxins such as herbarumin I [173], thaxtomin A (**35**) [174], radicinin [175].

Third, there is no sufficient information on the stability of natural phytotoxins and the general toxicity of their degradation products. Just a little can be found on mycotoxins or metabolites produced by microbial biocontrol agents [176,177,178,179,180].

Another problem that needs to be solved for the effective use of BCHs is the selection or development of NP formulations [172,181]. For the initial evaluation of BCHs, the simplest formulations are usually tested. The sorgoleone (**26**) prototypic formulation, which was prepared as wetting powder using spray drying, had the following composition: AI (4.9%), kaolin (79.2%), silicon dioxide (9.2%) and an adhesive (monooctadecyl ether of polyoxyethylene, 7.0%) [182]. For the application of phytotoxic culture fluid of the fungus *Phoma* sp., an emulsion based on Span-80, Tween-80, and palm oil was developed [183].

The data on the evaluation of mixtures of BCHs with chemical or biological herbicides are still not numerous. Some compounds are able to inhibit detoxifying plant enzymes, such as S-glutathione-transferase and monoxygenase inhibitors. Copper and zinc chelators inhibited the detox enzymes, synergizing paraquat, and other active oxygen-generating herbicides. For instance, fungal macrocidin A and some other phytotoxins (e.g., tenuazonic acid) are chelators and could be synergistic with paraquat [149,184]. Pretilachlor and sunflower leaf extract mixture was shown to be synergistic at the ratio 1:9 with higher phytotoxicity against *E. crus-galli* than the components separately [185]. Synergistic herbicidal action was demonstrated for water-soluble and volatile compounds from *Cytisus scoparius* (L.) Wimm. ex W.D.J. Koch foliage [186]. Sarmentosine from *Piper sarmentosum* Roxb. biomass exhibited synergistic effects against *E. crus-galli* and *A. retroflexus* when it was applied in combination with a commercial CH, propanil [187].

The adjuvant Hasten™ based on ethyl and methyl esters of vegetable oil significantly increased in vivo activity of the two fungal phytotoxins, phaeosphaeride A (**31**) and stagonolide A, produced by *Paraphoma* sp. and *Stagonospora cirsii*, respectively [188,189]. The prototypic BCH for field testing based on tenuazonic acid included 0.1% AI and 0.4% adjuvant based on polyoxyethylene ether of fatty alcohols and laurocarpam (1:3 by volume) [170]. The new formulation of strigolactones prepared as an emulsion concentrate reduced the germination of the *Striga* seeds by 89–99% in greenhouse experiments and was also effective in the field [190]. Solid formulation of culture fluid *Diaporthe* sp. obtained by spray drying was prepared with adjuvant AgRho FKC, which is known as a glyphosate activator, and silicon dioxide as a filler [191]. Inuloxin (**23**) encapsulated in cyclodextrin retained high activity against *Phelipanche ramosa* (L.) Pomel weed [192]. A biofilm based on polybutylene succinate was successfully tested, providing gradual release of an organic extract of *Dittrichia viscosa* (L.) Greuter [193]. The half-life of scopoletin (**13**) loaded in organoclay granules (Cloisite^®®^ 10A) was 20 days, in contrast to its free form, for which the half-life was less than a day. The solid formulation of this phytotoxin showed herbicidal activity in the field at the application rate of 12 kg/ha [194].

Nanotechnology could provide a new impulse in the enhancement of BCHs [195,196,197]. For instance, summer savory (*Satureja hortensis* L.) essential oil, which was nanoencapsulated in Persian gum, provided full control of *A. retroflexus* at a dose of 15 mL/L 48 h after the treatment [198]. However, ophiobolin A (**27**) encapsulated in mesoporous silica nanoparticles was not so effective in showing phytotoxicity for the leaves of various plants only when they were mechanically damaged [199]. Phytotoxic ailanthone (**25**) from *Ailanthus altissima* (Mill.) Swingle loaded into dextrin-based nanosponges displayed herbicidal action prolonged for several weeks [200]. Nanoemulsion based on citronella grass (*Cymbopogon nardus* Rendle) essential oil containing 36% geraniol, 18% *trans*-citral, and 15% *cis*-citral displayed a significant dose-dependent inhibitory effect on seed germination and seedling growth of *E. crus-galli* [201].

## 4. Natural Phytotoxins as Prototypes of Synthetic Herbicides

As mentioned above, there are various approaches to the formation of CH screening libraries. For this purpose, the employment of natural phytotoxins is rational since a significant proportion of them corresponds to the physico-chemical properties of herbicidal molecules and exhibits original MOA on plants [26,119]. For instance, NPs can be included for virtual and biological screening of inhibitors of known MTs, while having a new structural skeleton compared to CHs. For example, a virtual screening of 14 natural phytotoxins for binding to the D1 protein (*psbA*) of photosystem II of three weed species (*Eleusine indica* (L.) Gaertn., *Praxelis clematidea* (Hieron. ex Kuntze) R.M.King & H.Rob., and *Momordica charantia* L.) was conducted using the PyRx v.0.9.5 program. As a result, aurachin A and aurachin P, as well as cyanobacterin were placed in the top rating of compounds with a high-affinity index to the specified MT. The binding of the phytotoxins to protein D1 was determined by their interaction with certain amino acid residues, and the set of amino acids involved in binding turned out to be 50–90% similar to the one for its complex with diuron [202]. A complex-stage virtual screening of inhibitors of 5-enolpyruvylshikimate-3-phosphate synthase (EPSPS) was designed. First, a virtual library of herbicide-like NPs from various sources was established; after that, the substances were evaluated by modeling their interaction with EPSPS from *E. indica*. One of the compounds that passed the screening showed high affinity to EPSPS and formed a stable complex with the enzyme. Notably, its structure was not similar to glyphosate affecting EPSPS [203]. In a similar way, the established crystal structure of unique MTs of natural phytotoxins can facilitate the virtual search for herbicidal molecules from chemical libraries by molecular docking and further rational design of compounds with a simplified structure, increased activity, and low general toxicity. Despite the fact that more than 40 such MTs are known at the moment, this strategy has remained underexplored [119].

NPs and NP-derived CHs (both NP derivatives and NP mimic) account for about 7% of chemical weed control means. The share of CHs containing NP toxophores or fragments (NP synthetic equivalents) reaches 34% of the total amount of the first-in-class registered CHs [204]. For example, glufosinate (phosphinotricin) is a synthetic analog of the bacterial metabolite bialaphos mentioned above, which is metabolized in plants to phytotoxic phosphinotricin [205]. Such well-known auxin-like herbicides as clopyralid (**2**) and aminopyralid are synthetic equivalents of picolinic acid produced by soil and phytopathogenic fungi, such as *Aspergillus* spp. and *Pyricularia oryzae* [206]. Some modern HPPD inhibitors (mesotrione, sulcotrione, tembotrione, etc.) are synthetic equivalents of NPs from the triketone group (for example, leptospermone (**21**)) isolated from various plants of the *Myrtaceae* family [119]. Notably, the role of NPs in the development of insecticides and fungicides is much higher [204].

A complete synthesis of natural phytotoxins and, in particular, enantioselective synthesis could represent a way to create promising libraries of herbicidal compounds. However, NPs usually have a rather complex structure with several stereogenic centers, which makes it difficult to obtain them by synthetic methods on an industrial scale with high yields while using environmentally friendly reagents. This problem can be partially solved by searching and synthesizing simplified analogs of NPs [204]. For example, reduced active analogs of the fungal phytotoxins phyllostictin A [207], ophiobolin A [208], macrocidin A [209] were obtained.

**Figure 4:**
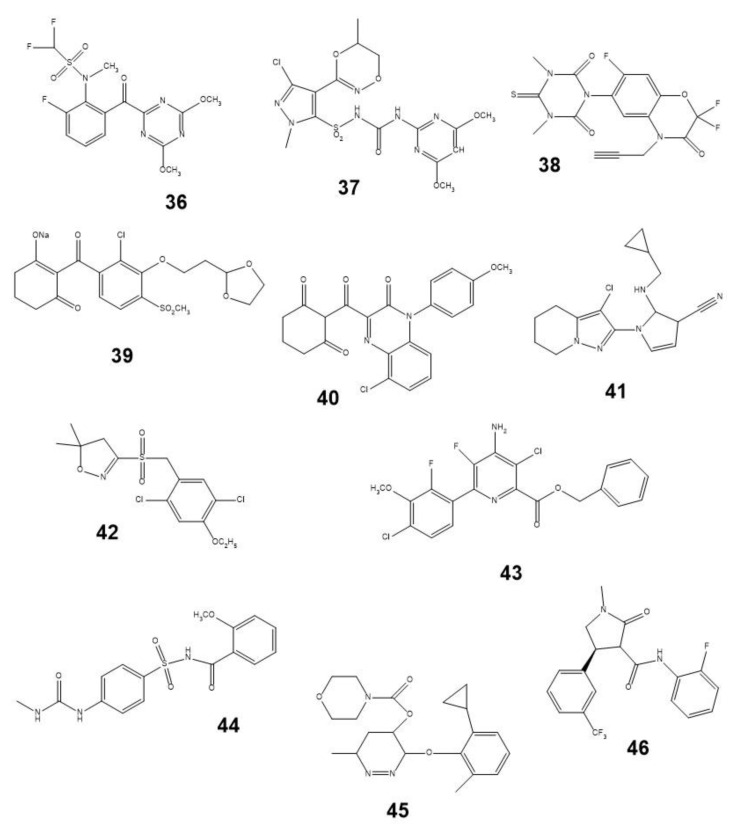
Examples of some new chemical herbicides: **36**—triafamone, **37**—metazosulfurone, **38**—trifludimoxacin, **39**—lancotrione-sodium, **40**—fenquinotrione, **41**—cyclopyranil, **42**—fenoxasulfone, **43**—metcamifen, **44**—florpyrauxifen-benzyl, **45**—cyclopyrimorate, and **46**—tetflupyrolimet.

When considering the structural features of some phytotoxins (Figure 3) and common (Figure 2) or recently introduced CHs (Figure 4), it is easy to notice their numerous differences. Obviously, the herbicidal properties of some natural phytotoxins could be improved by their structural modification in accordance with the physico-chemical rules established for CHs [15,22,23,210,211]. For example, more than 25% of known CHs contain fluorine atoms or trifluoromethyl groups [212,213]. Despite the fact that no herbicide was created by a simple modification of biotechnologically produced NPs, this strategy proved to be effective, for example, in the development of the semi-synthetic insecticide Inscalis^®®^ based on pyripyropene A produced by some soil fungi [214].

For the design and following synthesis of NPDs, the establishment of active centers in NP structures is of great importance. This task can be solved by analyzing the relationships between the structure and biological activity of the original NPs, and their natural and simplest semi-synthetic derivatives [215,216]. Structure-activity relationship (SAR) studies allow us to reveal approaches to the synthesis of equivalents and simplified analogs of NPs, as well as so-called pseudo-natural compounds [217,218,219]. Recently, a large number of SAR studies have appeared on the semi-synthetic derivatives of plant metabolites as potential CHs, for example, with maslinic acid [220], 2,4,5-trimethoxybenzaldehyde [221], coumarin [222], and berberine [223]. However, there are still relatively few papers on the SAR of microbial phytotoxins. In the major review [172], the SAR of six phytotoxins was considered: nonenolides, cytochalasines, as well as derivatives of chenopodolin, papiracillic acid, and agropyrenal. Recently published SAR papers concern radicinin [224], picolinic acid [225], fumonisin [226], and some 10-membered lactones [227] that may have potential as BCHs. After studying the reactivity of the scaffold NPs and identifying semi-synthetic lead compounds, their modification can further be focused on the improvement of physico-chemical properties of CHs and increased phytotoxicity with characteristic toxophores or structural fragments. The selection of promising molecules can be confirmed by molecular docking if their potential MT is known.

A series of derivatives of 3-acyl-5-alkyltetramic acid was generated in a simulation model of the molecular interaction between tenuazonic acid (TeA) and the target protein D1 of *A. thaliana*. Then, each derivative that demonstrated high affinity to the protein was subjected to molecular docking to calculate the free binding energy. Three virtually selected TeA derivatives—D6 (*sec*-pentyl-TeA), D13 (*sec*-hexyl-TeA), and D27 (*sec*-heptyl-TeA)—were synthesized for bioassay. Among them, D6 and D13 showed higher herbicidal activity than TeA [228].

Plant species of the genus *Peperomia* produce a wide range of secondary metabolites. For instance, they are an important source of 2-acylcyclohexane-1,3-diones (for example, alatanone A (**17**), trineuron A, etc.) structurally similar to leptospermone and commercial triketone herbicides, such as sulcotrione. Seventy-six analogs of 2-acylcyclohexane-1,3-dione were synthesized, which were tested for the inhibition of 4-hydroxyphenylpyruvate dioxygenase (HPPD). The resulting data set was subsequently analyzed using a three-dimensional quantitative SAR to characterize the key structural features that contribute to the inhibition of HPPD activity. This revealed 2-acylcyclohexane-1,3-dione with a side C11 alkyl chain which demonstrated higher herbicidal activity than sulcotrione [229].

A plant growth inhibitor, xanthoxyline (**14**), is a small natural methyl ketone from the plant *Zanthoxylum limonella* (Dennst.) Alston. In the study [230], related methyl ketones carrying electron-donor and acceptor groups or heterocyclic substituents were investigated as the inhibitors of seed germination and growth of seedlings of *Amaranthus tricolor* L. and *E. crus-galli*. On the SAR basis, the types and positions of substituents that are crucial for the activity of methyl ketone herbicides were established. It was found that indole derivatives, namely 3-acetylindole and 3-acetyl-7-azaindole, are the most active methyl ketones that strongly inhibit plant growth at low concentrations. The molecular docking results indicated the carbonyl, aromatic, and azaindole groups to be important for the interaction of the selected methyl ketones with plant HPPD [230].

## 5. Conclusions

The analysis of the literature shows that the progress in the development of natural products for weed control follows the general advent of the exploration of synthetic herbicides. The virtual search for herbicide-like natural phytotoxins and their evaluation with the bioassays accepted in the screening of herbicidal molecules should increase the selection efficacy. Biotechnological optimization of the production and careful toxicological characterization will facilitate unmodified NPs to be more attractive for practice. The development of stable formulations of NPs providing improved leaf penetration and controlled release in soil may reduce their application rates and environmental risks. The defined MOAs of herbicidal NPs will serve as a scientific base for the composition of synergistic mixtures of natural and synthetic herbicides. The crystal structures of MTs ascertained for natural herbicidal compounds may be used for the virtual screening of more effective synthetic inhibitors from existing chemical libraries. The computer-aided design of NP derivatives using CH-specific toxophores and physico-chemical rules combined with structure–activity relationship analysis will allow us to find hits for the development of NP-derived CHs. All these approaches should potentially accelerate the appearance of novel herbicides so wanted in both organic and conventional agriculture.

## Data Availability

Not applicable.
